# Latest updates on *MET* targeted therapy for EXON 14 mutations in lung cancer

**DOI:** 10.18632/oncotarget.28419

**Published:** 2023-05-26

**Authors:** Mira Al Jaberi, Wolfgang Clough, Samir Dalia

**Keywords:** non small cell lung cancer, MET mutation, targeted therapy, precision medicine

Several alterations in the MET gene were identified as targetable oncogenic changes leading to non-small cell lung cancer (NSCLC). These include genomic amplifications, exon 14 skipping mutations and fusion [1, 2]. Capmatinib has been considered as a first-line treatment for patients with NSCLC carrying a *MET* exon 14 skipping mutation since May 2020 by the USFDA [3]. A study newly published in early 2023 showed that Crizotinib; a tyrosine kinase inhibitor was also effective for *MET* fusions, which occur rarely in 0.2–0.3% of patients with lung cancer [1].

A major challenge arising after the introduction of tyrosine kinase inhibitors is limited clinical benefit, which is due to primary and potential secondary acquired drug resistance [4, 5]. Several structurally different *MET* tyrosine kinase inhibitors (TKIs) have been developed or are under clinical evaluation. TKIs are categorized into type I TKIs (type Ia: crizotinib; type Ib: savolitinib, capmatinib) and type II TKIs (cabozantinib, glesatinib, merestinib). Combination therapy reduces resistance and enhances clinical outcomes [5]. A clinical study showed that combinations of type I/II TKI inhibitors (capmatinib and merestinib) yielded no resistant clones *in vitro* and led to a significant reduction in tumor outgrowth *in vivo* compared to either MET inhibitor alone [5]. In addition, one study showed that in general, type Ib inhibitors were more unlikely to develop resistance than type II inhibitors [6]. Furthermore, the efficacy of resistance suppression was inversely correlated with drug concentration, where greater secondary mutations emerged at lower drug concentrations [5, 6]. This highlights the importance that patients should be on FDA approved drug dosing (e.g., Capmatinib 400 mg orally twice daily) as it is unclear if lower doses have efficacy currently [3].

Based on ongoing Phase I CHRYSALIS, Amivantamab; fully human bispecific antibody targets both *EGFR* and *MET*. It has shown promising results against NSCLC patients with *EGFR* 20 insertion and NSCLC patients with *MET* exon 14 skipping mutations. The drug is given as 1050 mg (pts <80 kg) or 1400 mg (pts ≥80 kg) once weekly in cycle 1 and twice a week until disease progression. This study included 43 pts with *MET* exon 14 skipping mutations. Overall response rate was 33%. The study is still ongoing and results are promising [7].

Other Phase I/II study conducted in China, assesses the use of APL-101 a novel, potent, selective c-MET inhibitor, in patients with NSCLC and advanced malignancies with c-Met dysregulation. Study is still ongoing and results are not yet available [8].

These clinical trials along with others will show us if other MET inhibitors or combination therapy may be better than the current standard of care. The future looks bright for patients with MET mutations and NSCLC.
